# Human *in vivo* medial gastrocnemius gear during active and passive muscle lengthening: effect of inconsistent methods and nomenclature on data interpretation

**DOI:** 10.1242/bio.060023

**Published:** 2023-09-05

**Authors:** Matheus Daros Pinto, Kazunori Nosaka, James M. Wakeling, Anthony J. Blazevich

**Affiliations:** ^1^ School of Medical and Health Sciences, Edith Cowan University, Joondalup 6027, Australia; ^2^ Department of Biomedical Physiology and Kinesiology, Simon Fraser University, Burnaby, V5A 1S6, Canada

**Keywords:** Muscle architecture, Ultrasound, Isokinetic, Fascicle length, Belly gear, Architectural gear ratio (AGR)

## Abstract

**‘**Muscle gear’ is calculated as the ratio of fascicle-to-muscle length change, strain, or velocity. Inconsistencies in nomenclature and definitions of gear exist across disciplines partly due to differences in *fascicle* [curved (*L_f_*) versus linear (*L_f,straight_*)] and *muscle* [whole-muscle belly (*L_b_*) versus belly segment (*L_b,segment_*)] length calculation methods. We tested whether these differences affect gear magnitude during passive and active muscle lengthening of human medial gastrocnemius of young men (*n*=13, 26.3±5.0 years) using an isokinetic dynamometer. *L_b_*, *L_b,segment_*, *L_f_* and *L_f,straight_* were measured from motion analysis and ultrasound imaging data. Downshifts in belly gear but not belly segment gear occurred with muscle lengthening only during active lengthening. Muscle gear was unaffected by fascicle length measurement method (*P*=0.18) but differed when calculated as changes in *L_b_* or *L_b,segment_* (*P*<0.01) in a length-dependent manner. Caution is therefore advised for the use and interpretation of different muscle gear calculation methods and nomenclatures in animal and human comparative physiology.

## Teaser

**Summary:** We identified inconsistencies in muscle gearing nomenclature and calculation methods across the biological sciences. Our empirical data show that these methodological differences affect conclusion depending on the contraction mode used and is thus important in animal and human comparative physiology.

## INTRODUCTION

When pennate muscles develop active force, fascicles change in both length and angle. Fascicle rotation decouples the muscle belly length change from longitudinal fascicle length change, effectively allowing a muscle to operate in a gear that varies in response to the task's mechanical demands (Azizi et al., 2008; Dick and Wakeling, 2017; Eng et al., 2018; Holt et al., 2016). This muscle-to-fascicle length change ratio has been variably referred to as the *architectural gear ratio* (AGR), *belly gear*, or *muscle gear* (Azizi et al., 2008; Azizi and Roberts, 2014; Holt et al., 2016; Randhawa and Wakeling, 2013; Wakeling et al., 2011).

**Fig. 1.**
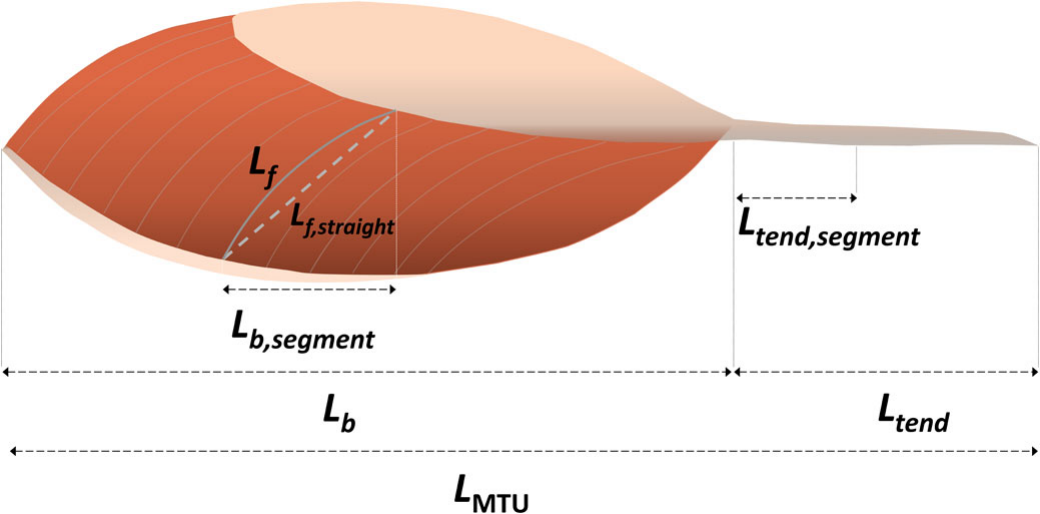
Schematic of anatomical inputs used to calculate muscle gear: fascicle length (*L_f_*), fascicle length (*L_f,straight_*), belly segment length (*L_b,segment_*), whole muscle belly length (*L_b_*), and tendon length (*L_tend_*), tendon segment length (*L_tend,segment_*), and muscle-tendon unit length (*L*_MTU_).

In the early 2000s, AGR was first proposed as a method to understand the mechanics of segmented musculature during salamander locomotion and was defined as the ratio of longitudinal muscle segment strain to the fibre strain within that segment (Brainerd and Azizi, 2005). Later, Azizi et al. (2008) defined AGR as the ratio of whole muscle belly or muscle-tendon unit (MTU) to fibre-shortening velocities. Several studies in vivo subsequently used a mixed definition, labelled ‘belly gear’, which was defined as the ratio of the muscle segment to fascicle velocities (Monte et al., 2021; Randhawa and Wakeling, 2013; Wakeling et al., 2011). This was determined from the segment of the muscle that was encompassed by the fascicle itself using simple trigonometry (*L*_*b*,*segment*_=*L*_*f*_×cos*θ*; Fig. 1) and is fundamentally different from the AGR first used by Brainerd and Azizi (2005) because fascicle or fibre lengths and velocities rather than strains were measured. These differences in mathematical computation (strain versus length change) and the anatomical inputs used to calculate muscle gear (whole muscle versus segment) may provide different gear ratios and lead to incorrect qualitative interpretations. It may additionally confound the comparison of outcomes between studies investigating gear differences across muscles, animal species, and contraction conditions, or with exercise training, ageing, and disuse.

In the present paper, our first aim is to outline current inconsistencies in nomenclatures and calculation methods. We provide a schematic of the anatomical inputs generally used to calculate ‘muscle gear’ and summarise relevant studies reporting calculations and the nomenclatures used (Table 1). Our second aim is to assess whether different muscle gear calculation methods affect the information obtained from experiments. We tested this under varying eccentric muscle contraction conditions because substantial interest in eccentric contractions currently exists across disciplines but is relatively understudied. We used two base definitions according to whether the belly segment length (encapsulated by the fascicle that is measured; *L_b,segment_*) or the whole muscle belly length (*L_b_*) is calculated:

1. Belly Segment Gear: Δ*L_b,segment_*/Δ *L_f,straight_*
2. Belly Gear: Δ*L_b_*/Δ*L_f_*

**Table 1.**
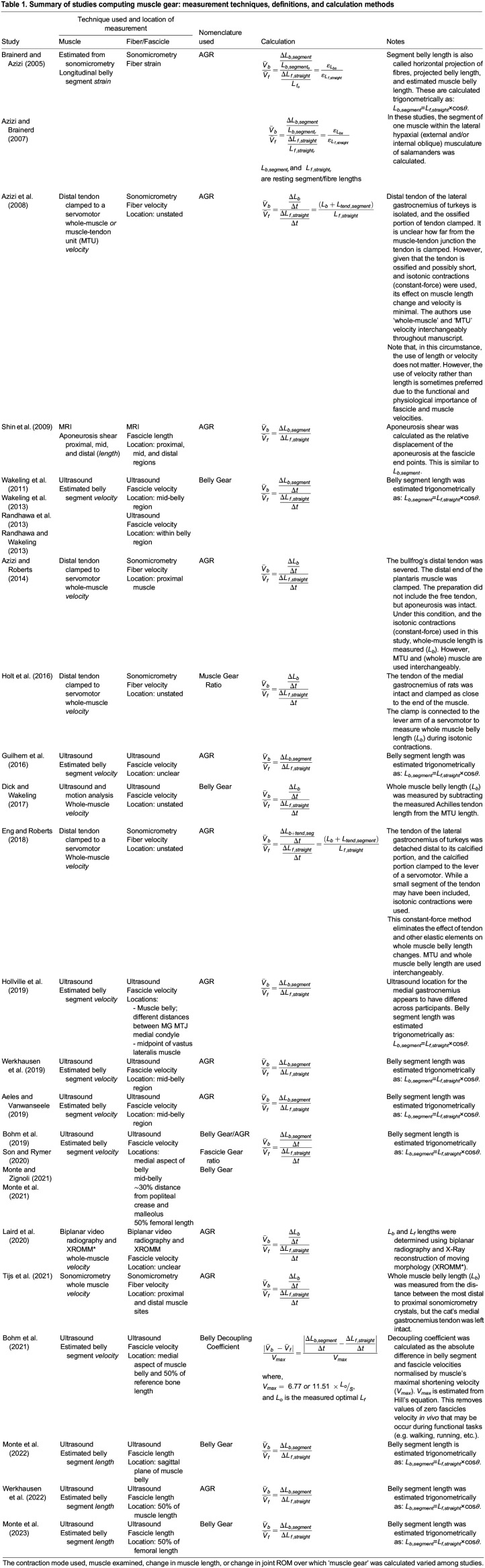
Summary of studies computing muscle gear: measurement techniques, definitions, and calculation methods

Two factors might influence the muscle gear calculation. First, the choice of fascicle length measurement method. In many studies, fascicles have been assumed to run linearly between origin and insertion (measured as the ‘straight fascicle length’, *L_f,straight_*), however fascicles in many muscles follow a curved path (‘curved fascicle length’, *Lf*) and are susceptible to changes in curvature during contraction (Muramatsu et al., 2002; Namburete et al., 2011; Van Leeuwen and Spoor, 1992). The extent to which the assumption of fascicle path linearity affects gear calculation has not been investigated. A second possible factor is whether Δ*L_b_* or Δ*L_b,segment_* (or strain) is used. Δ*L_b_* and Δ*L_b,segment_* will differ if regions of a muscle vary in their length changes or if aponeuroses lengths change during contraction. Alternatively, significant differences in fascicle orientation and rotation along the muscle could also affect Δ*L_b,segment_* because it is mathematically dependent on (the cosine of) fascicle angle. In the human medial gastrocnemius (MG), for example, fascicle lengthening during eccentric contractions are relatively uniform along the muscle while fascicle rotation is greater in proximal than distal segments (Shin et al., 2009). Thus, Δ*L_b,segment_* must differ at proximal and distal sites of the muscle, and it is likely that the Δ*L_b,segment_* recorded in some of its parts may not reflect overall Δ*L_b_* changes. Because most studies in humans have used sonographic recordings at a single muscle belly location (i.e. *L_b,segment_*), it is unlikely that results can be extrapolated between studies using different methods or where images were recorded at different locations within the muscle.

The overall aim of the present study, therefore, was to determine whether different gear calculation methods (*L_f_* versus *L_f_,_straight_* and *L_b_* versus *L_b,segment_*; length changes versus strains) provide comparable outcomes and conclusions under different muscle lengthening contractile conditions (relaxed versus active). MG was chosen for study as it is commonly used in gearing studies in humans (Dick and Wakeling, 2017; Monte et al., 2021; Randhawa et al., 2013; Randhawa and Wakeling, 2013; Wakeling et al., 2011), the fascicles are relatively short and can thus be measured accurately, and fascicles tend to be approximately straight during relaxed stretches yet substantially curved during active contraction, allowing the effects of fascicle curvature to be assessed.

## RESULTS

### Effect of fascicle length calculation method: curved versus straight fascicle

**Fig. 2.**
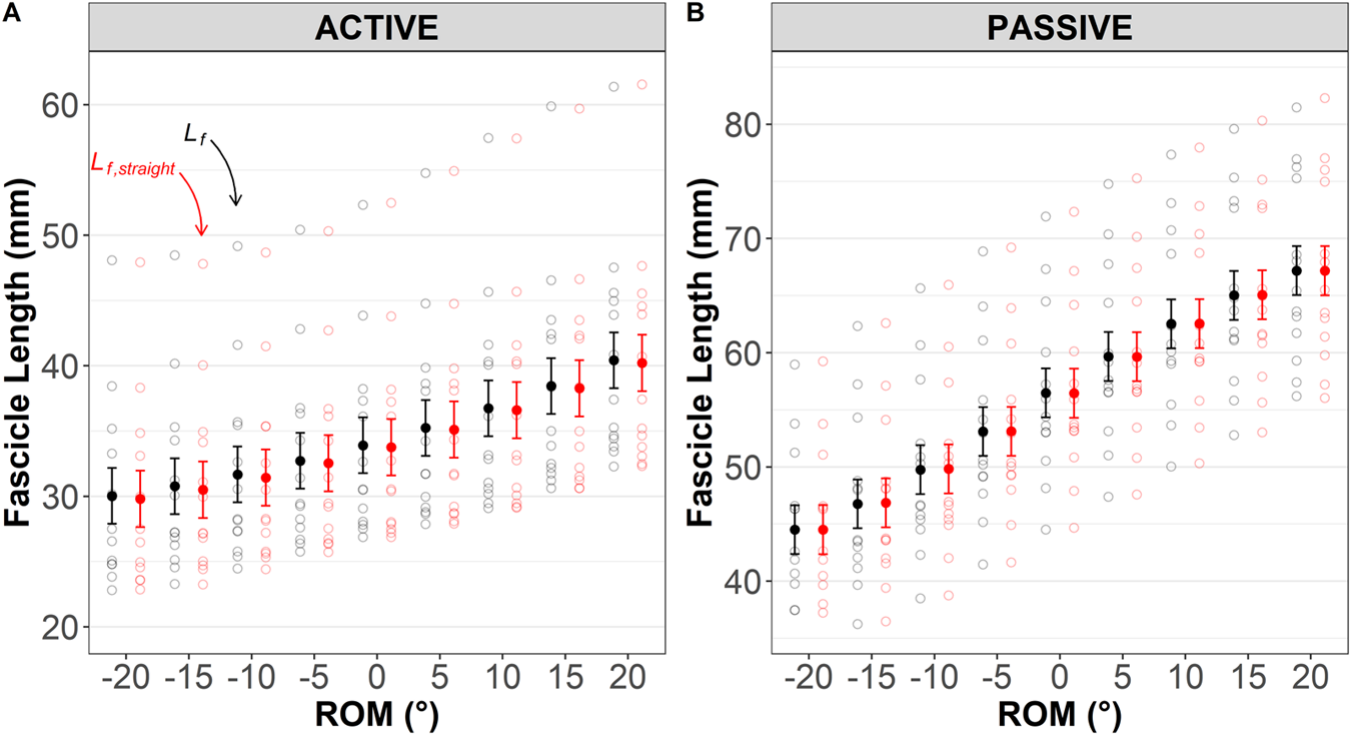
**Medial gastrocnemius fascicle length measured directly [assuming curvature (*Lf*); *black*] and calculated trigonometrically [assumed straight (*Lf,_straight_*); *red*] during (A) active (maximal eccentric) and (B) passive (relaxed muscle stretch) tests.** Results are displayed as predicted values of fascicle length (conditional effects) and the level of confidence in the mean is indicated by the standard error. Different y-axis scales are used for the purpose of visualization.

Active lengthening (maximal eccentric contraction): no method×ROM interaction (*P*=1.0) or effect of method were detected (*P*=0.16), suggesting a lack of effect of the use of curved versus straight fascicle lengths [mean difference=0.19 (95% CI: −0.10–0.50)] on the mean fascicle length, as shown in Fig. 2A.

Passive lengthening (relaxed muscle stretch): no method×ROM interaction (*P*=1.0) or effect of method were detected (*P*=0.88), suggesting no effect of use of curved versus straight fascicle lengths [mean difference=−0.02 (−0.32–0.28)] on the mean fascicle length, as shown in Fig. 2B.

### Effect of muscle length calculation method: belly segment length (L_b,segment_) versus whole muscle belly length (L_b_) displacements

**Fig. 3.**
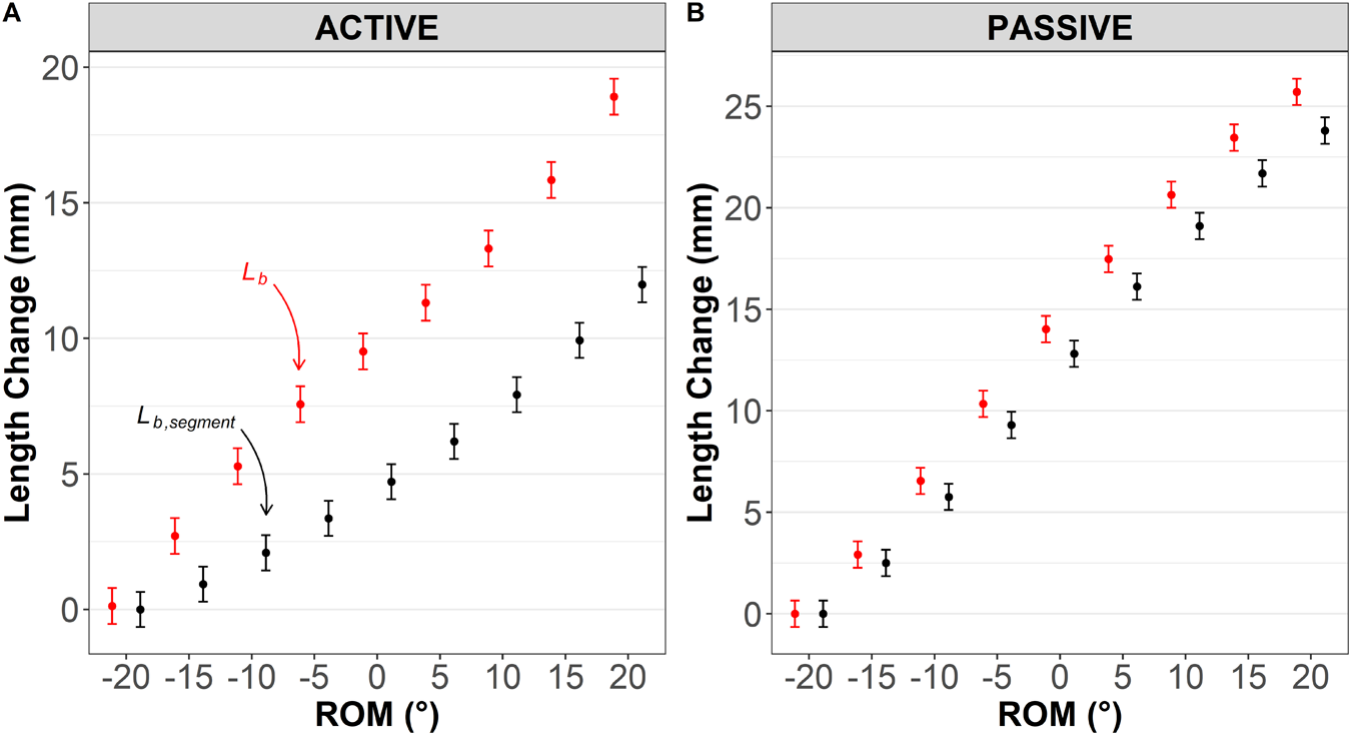
**Changes in medial gastrocnemius segment (*L_b,segment_*; black) and whole muscle belly (*L_b_*; red) lengths during (A) active (maximal eccentric) and (B) passive (relaxed muscle stretch) tests.** Results are displayed as predicted values of *L_b_* and *L_b,segment_* length (conditional effects) and the level of confidence in the mean is indicated by the standard error. Note that different y-axis scales are used for the purpose of visualization.

Active lengthening: a significant method×ROM interaction (*P*<0.001) and main effect of method were detected (*P*<0.001). Further analysis revealed significantly greater *L_b_* than *L_b,segment_* change [mean difference=4.16 (3.72–4.60), *P*<0.001]. As shown in Fig. 3A, *L_b_* change was significantly greater than *L_b,segment_* from 15° of plantarflexion [mean difference=1.78 (0.47–3.08), *P*=0.008] and remained significantly greater over the rest of the ROM. *L_b_* change at 20° dorsiflexion was significantly greater [18.9 (17.6–20.3)] than *L_b,segment_* [12.0 (10.7–13.3)] with a mean difference of 6.93 (5.62–8.24), *P*<0.001].

Passive lengthening: no method×ROM interaction was detected (*P*=0.52) but a main effect of method was detected (*P*<0.001), suggesting an overall effect of muscle length calculation method. Analysis revealed significantly greater *L_b_* than *L_b,segment_* change [mean difference=1.12 (0.68–1.55), *P*<0.001], as shown in Fig. 3B.

### Effect of fascicle and muscle length calculation methods on fascicle and muscle strains

**Fig. 4.**
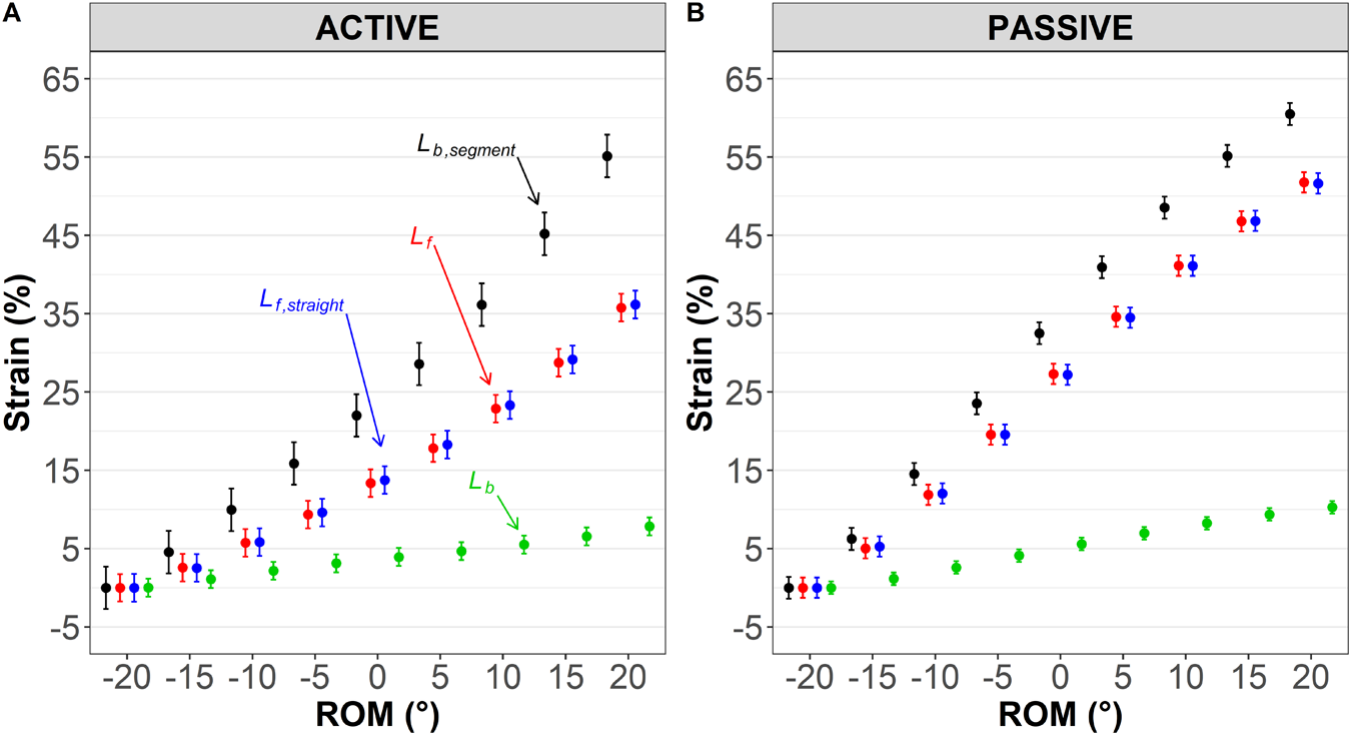
**Effect of medial gastrocnemius fascicle and muscle length input methods (straight versus curved and segment versus whole muscle) on linear fascicle (*L_f,straight_*; *blue*), measured fascicle (*L_f_*; *red*), whole muscle belly (*L_b_*; *green*), and belly segment (*L_b,segment_*; *black*) strains (% relative to initial length) during (A) active (maximal eccentric) and (B) passive (relaxed muscle stretch) tests.** Results are displayed as predicted values of fascicle and muscle strains (conditional effects) and the level of confidence in the mean is indicated by the standard error.

Active lengthening: a significant method×ROM interaction (*P*<0.001) and main effect of method were detected (*P*<0.001). Further analysis revealed significantly greater *L_b,segment_* than *L_b_* strain [mean difference=20.3 (13.3–27.3), *P*<0.001]. As shown in Fig. 4A, *L_b,segment_* was significantly greater than *L_b_* strain over the first 10° of plantarflexion [mean difference=7.8% (0.10–15.5), *P*=0.046] and remained significantly greater over the rest of the ROM (*P*<0.001). The strain from −20° plantarflexion to 20° dorsiflexion was significantly greater at the *L_b,segment_* [55.1% (49.4–60.9)] than *L_b_* strain [7.84% (5.58–10.1)] with a mean difference of 47.3% (39.6–55.0, *P*<0.001). No significant differences in mean strains were detected between *L_f_* and *L_f,straight_* [mean difference=−0.27 (−1.80–1.26), *P*=0.63]. The average strains from −20° plantarflexion to 20° dorsiflexion for *L_f_* and *L_f,straight_* were 35.8% (32.2–39.40) and 36.2% (32.5–39.8), respectively.

Passive lengthening: a significant method×ROM interaction (*P*<0.001) and main effect of method were observed (*P*<0.001). Further analysis revealed significantly greater *L_b,segment_* than *L_b_* strain [mean difference=26.0 (22.4–29.5), *P*<0.001]. As shown in Fig. 4B, *L_b,segment_* strain was significantly greater than *L_b_* strain over the first 5° of plantarflexion [mean difference=5.1% (0.79–9.38), *P*=0.015] and remained significantly greater over the rest of the ROM. The strain from −20° plantarflexion to 20° dorsiflexion was significantly greater at the *L_b,segment_* [60.5% (57.6–63.4)] than *L_b_* strain [10.3% (8.70–11.9)] with a mean difference of 50.2% (45.9–54.5, *P*<0.001). No significant difference in mean strains were detected between *L_f_* and *L_f,straight_* [mean difference=−0.01 (−1.13–1.11), *P*=1.0]. The average strains from −20° plantarflexion to 20° dorsiflexion for *L_f_* and *L_f,straight_* were 51.8% (49.1–54.4) and 51.6% (49.0–54.3), respectively.

### Effect of muscle gear calculation method (belly gear, belly segment gear, and modified belly segment gear)

A significant method×condition×ROM interaction was detected (*P*<0.001), suggesting that the effect of muscle gear calculation method was affected by contraction condition and ROM.

Active versus passive lengthening: belly gear was significantly greater during active than passive lengthening [mean difference=0.73 (0.54–0.92), *P*<0.001], but no differences in belly segment gear or modified belly segment gear between active and passive trials were detected [mean differences=0.10 (−0.09–0.28) and 0.13 (−0.06–0.31), *P*≥0.41], suggesting that between-condition differences in gear calculation are method dependent. Differences in belly gear between active and passive trials were only detected at −10° and −5° plantarflexion [mean differences: 1.89 (1.46–2.32) and 0.92 (0.48–1.35), *P*<0.0001, respectively].

**Fig. 5.**
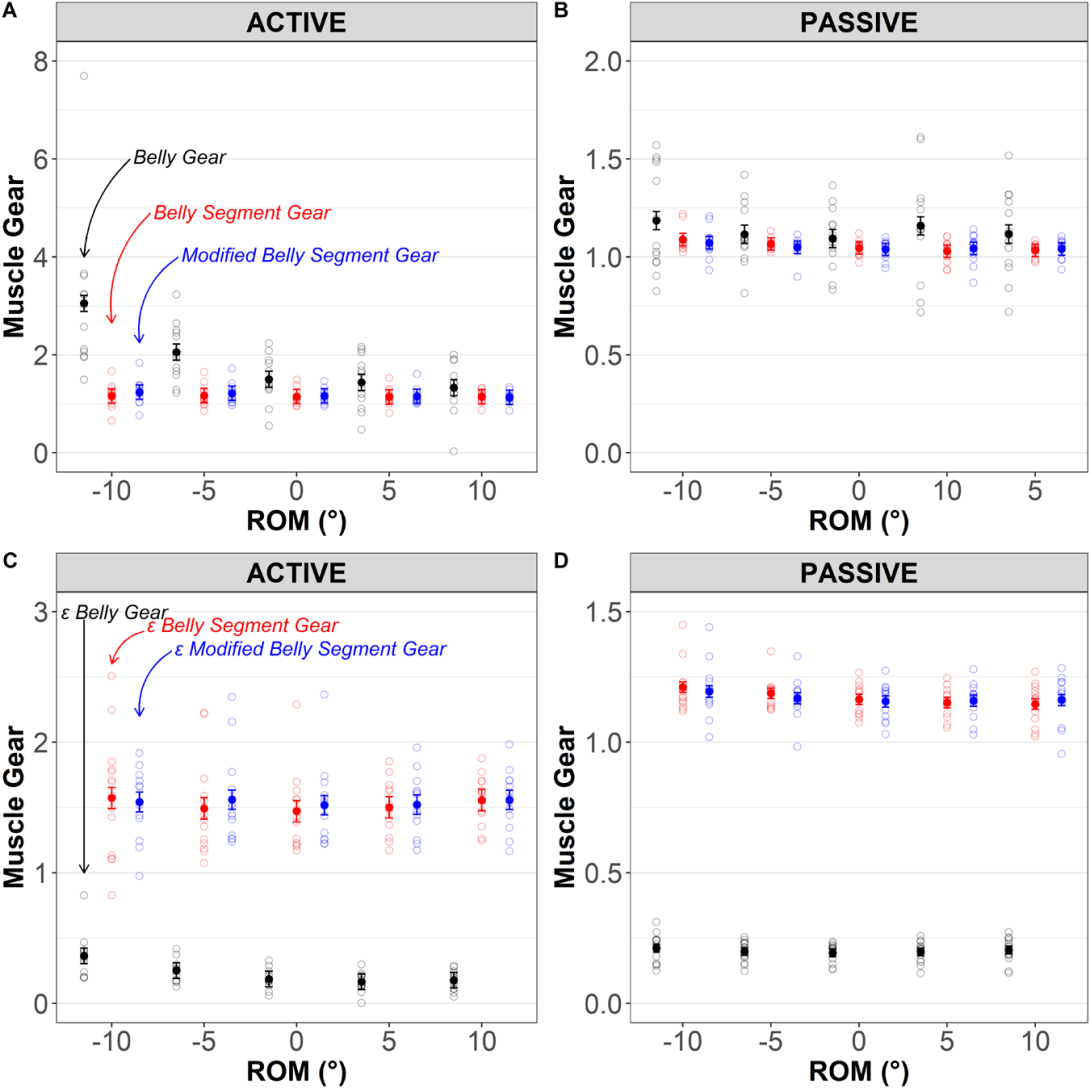
**Effect of muscle gear calculation methods over the range of motion (ROM).** Top panels illustrate data using raw displacements, whereas bottom panels report strain (ε) values. Panels A and C represent muscle gearing during active contraction (maximal eccentric), whereas panels B and D represent passive (relaxed muscle stretch) tests. In the top panels, muscle gear was determined using belly gear (black), belly segment gear (red), modified belly segment gear (blue). In the bottom panels, muscle gear was determined using their strain (ε) extensions: εbelly gear (black), εbelly segment gear (red), and εmodified belly segment gear (blue); see text or Table 2 for details. Muscle gear ratios were calculated for each 10° of the ROM using first central difference method (e.g. length change from −15 to −5, −10 to −5, etc). Results are displayed as predicted values of muscle gear (conditional effects) and the level of confidence in the mean is indicated by the standard error. Open circles indicate individual (raw) data. Belly gear and εBelly gear raw data for active contractions are shown for 12 subjects because ultrasound videos captured at the MTJ were corrupted in one individual. Different scales are used for the purpose of visualization.

Effect of gear calculation method during active lengthening: belly gear was significantly greater than belly segment gear [mean difference: 0.72 (0.41–1.03), *P=*0.0002] and modified belly segment gear [mean difference=0.69 (0.39−1.00), *P=*0.0002] but no differences between belly segment gear and modified belly segment gear were detected [mean difference=−0.03 (−0.28–0.22), *P*=0.95]; i.e. the assumption of fascicle linearity had no effect. As shown in Fig. 5A, belly gear was significantly greater than belly segment gear at −10° and −5° plantarflexion [mean differences: 1.89 (1.37–2.40), *P*<0.001, and 0.92 (0.40–1.43), *P*<0.0001, respectively]. Similarly, belly gear was significantly greater than modified belly segment gear at −10° and −5° plantarflexion [mean differences: 1.83 (1.32–2.35), *P*<0.001, and 0.87 (0.36–1.39), *P*<0.0001, respectively].

**Table 2.**
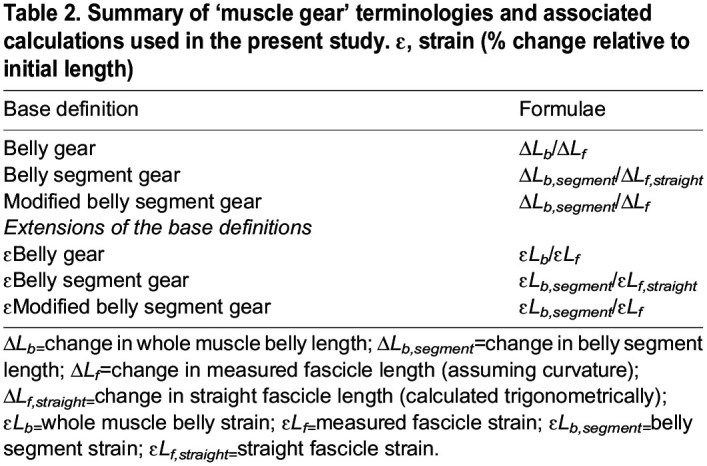
Summary of ‘muscle gear’ terminologies and associated calculations used in the present study. ε, strain (% change relative to initial length)

Changes in gear during active lengthening: belly gear was significantly reduced from −10° to −5° [mean difference: 1.0 (0.52–1.47), *P*<0.001] and from −5° plantarflexion to 0° [mean difference: 0.56 (0.08–1.03), *P*=0.009]. However, no changes in belly gear were observed from 0° to 5° or 10° dorsiflexion (*P*>0.99). Belly segment gear and modified belly segment gear did not change significantly during active lengthening (*P*>0.99).

Effect of gear calculation method during passive lengthening: no method×ROM interaction (*P*=0.94) or effect of method (*P*=0.075) were detected, suggesting a lack of effect of gear calculation method on muscle gear, as shown in Fig. 5B. The mean differences between belly gear and belly segment gear and belly gear and modified belly segment gear were 0.081 (−0.02–0.18), *P*=0.11, and 0.085 (−0.01–0.18), *P*=0.08, respectively.

Changes in gear during passive lengthening: no significant changes in belly gear, belly segment gear, or modified belly segment gear were detected during passive lengthening (*P*>0.51).

### Effect of muscle gear calculation method (εbelly gear, εbelly segment gear and εmodified belly gear calculated from strain values)

A significant method×condition interaction (*P*<0.001) was detected, suggesting that that the effect of muscle gear calculation method varied by contraction condition. However, no method×condition×ROM, method×ROM, or contraction×ROM interactions were observed (*P*≥0.66), suggesting that the effect of muscle gear calculation method was not affected by ROM.

Active versus passive lengthening: εbelly segment gear and εmodified belly segment gear were significantly greater in active than passive trials [mean difference=0.34 (0.25–0.42), *P*<0.001, and 0.38 (0.30–0.47), *P*<0.001, respectively], but no difference in εbelly gear between active and passive trials were detected [mean differences=0.03 (−0.06–0.11), *P*=0.94], suggesting that between-condition differences in gear calculation are method dependent.

Effect of gear calculation method during active lengthening: a significant effect of method was observed (*P*<0.001) such that εbelly segment gear [1.52 (1.38–1.66)] and εmodified belly segment gear [1.54 (1.43–1.66)] were greater than εbelly gear [0.23 (0.17–0.29)], as shown in Fig. 5C. The mean difference between εbelly gear and εbelly segment gear was −1.29 (−1.48 – −1.10), *P<*0.001, and between εbelly gear and εmodified belly segment gear was −1.31 (−1.48 – −1.15), *P<*0.001. No difference in εbelly segment gear and εmodified belly gear was detected [mean difference: −0.02 (−0.12–0.08), *P*=0.85].

Effect of gear calculation method during passive lengthening: a significant effect of method was observed (*P*<0.001) such that εbelly segment gear [1.17 (1.14–1.21)] and εmodified belly segment gear [1.17 (1.13–1.21)] were greater than εbelly gear [0.20 (0.18–0.22)], as shown in Fig. 5E. The mean difference between εbelly gear and εbelly segment gear was −0.97 (−1.01 – −0.93), *P<*0.001, and between εbelly gear and εmodified belly segment gear was −0.97 (−1.01 – −0.92), *P<*0.001. No difference in εbelly segment gear or εmodified belly segment gear was detected [mean difference: 0.004 (−0.018–0.027), *P*=0.48].

## DISCUSSION

Over the last ∼20 years, various nomenclatures and calculation methods have been used to describe a muscle's ‘gear’, and substantial methodological differences and reporting inconsistencies can be observed across disciplines (see Table 1). To fulfil Aim 1, we have summarised the relevant studies reporting gear calculations and the nomenclatures used is presented in Table 1 and provide a schematic of the anatomical inputs typically used to calculate ‘muscle gear’ in Fig. 1.

Our second aim was to determine whether methodological differences affect either the numerical outcomes or the conclusions that would be drawn from them. Muscle gear is mathematically dependent upon both fascicle and muscle length changes (or velocities/strains). In both animal and human research, fascicle lengths are often measured as a straight line from origin to insertion using sonomicrometry or ultrasonography. However, fascicles may follow a non-linear path during contraction as intramuscular pressure rises (Muramatsu et al., 2002; Namburete et al., 2011; Van Leeuwen and Spoor, 1992). In the human MG, Muramatsu et al. (2002) showed that fascicles were ∼6% longer during isometric contraction when estimated along their curved path. Here, MG was lengthened both passively and during maximal contraction, so the muscle operated eccentrically, and fascicle curvature was visually evident. However, while mean fascicle lengths were longer when measured along their curved path than when estimated as a straight line (Fig. 2), no statistical mean differences between *L_f_* and *L_f,straight_* or their changes in either absolute or strain magnitudes were detected likely because changes in curvature during contraction were minimal, thus curvature itself had little effect. Consequently, fascicle length measurement method did not affect the muscle gear calculation, as evidenced by the lack of difference between belly gear and modified belly gear ratios (Fig. 5A and B, top panel). While these results suggest that (not) accounting for fascicle curvature has negligible effect on fascicle length(ening) and muscle gear values under the present experimental conditions, it remains to be determined whether the results are replicable in other muscles, contraction modes, or even to other regions within MG, in which curvature or its variations during contraction may differ (Muramatsu et al., 2002).

A second factor potentially affecting muscle gear calculation is whether Δ*L_b_* or Δ*L_b,segment_* are used. The latter is easier to capture because it only requires a single ultrasound device (in humans) or sonomicrometry (in animals) region along with use of simple trigonometric calculations to compute. However, to compute belly gear in vivo in humans, a second ultrasound device placed over the distal muscle–tendon junction and a method of computing the distance between the muscle origin and the ultrasound probe (usually done using motion analysis methods) are needed to compute Δ*L_b_*, as used in the present study and others (Dick and Wakeling, 2017; Kay et al., 2016). Δ*L_b_* during both active and passive muscle lengthening were greater than Δ*L_b,segment_*, but the differences were larger in the active lengthening (Fig. 3). Consequently, muscle gear calculations were affected by the input values used and this difference varied both with muscle length (i.e. ankle joint angle; see below) and between contraction conditions. For example, belly gear was greater than belly segment gear during active (Δ=0.73) but not passive lengthening (Δ=0.10) suggesting that between-condition differences in gear calculation are method dependent (Fig. 5A and B, respectively). These results were detected in the human MG during active lengthening. It is unclear whether they also extend to animal models that often display smaller differences in the magnitudes of *L_b_* and *L_b,segment_* (and their changes) or tendon tissue compliance. However, it should be noted that even the small-volume MGs of Sprague–Dawley rats show distinct fascicle angles and rotations (and thus muscle gears) between proximal and distal regions during active shortening contraction, suggesting that *L_b_* and *L_b,segment_* and therefore belly and belly segment gears, should also differ (see discussion below). It would be interesting to examine whether these differences are also detected in other species and contraction types.

In the present study, we observed differences in the mean belly gear and belly segment gear over the eccentric contraction (∼1.9 versus ∼1.2), suggesting that the choice of gear calculation method affected the outcome. It is unclear whether such results would be found for other contraction modes, and this should be examined in future research. The observed differences were also impacted by joint range of motion. Specifically, differences in belly gear and belly segment gear were greater at short than long muscle lengths (plantar- than dorsiflexion; Fig. 5A), with clear differences identified at −10° and −5° of plantar flexion (Δ=1.89 and 0.92, respectively). Muscles worked at a high gear at shorter lengths (∼3.1) but at lower gear at longer lengths (∼1.3) during active lengthening, and therefore *belly gear* downshifted during the contraction. However, a different conclusion was drawn from analyses of *belly segment gear*, where mean gear was lower and gear shifts were absent, i.e., gear remained ∼1.2 over the full range. These method-dependent outcomes indicate caution when interpreting and comparing results among studies using different muscle gear calculation methods. Additionally, downshifts in belly gear suggest a muscle length- or joint angle-dependence of gear. While researchers have often reported the average gear across the contraction, reporting belly gear at specific muscle lengths or joint angles is an interesting approach that may best reflect changes in fascicle rotation during contraction. Thus, method choices should be strongly considered in future research aiming to quantify muscle gear (and its changes) between muscles or across contraction conditions of different animal species as well as when quantifying its temporal change with exercise training, ageing, and disuse.

Researchers often assume that Δ*L_b,segment_* are uniform across the muscle and that the muscle's aponeuroses are non-compliant and therefore that Δ*L_b,segment_* is a valid proxy for *L_b_*. Our findings do not support this assumption during maximal eccentric contractions since differences in Δ*L_b,segment_* and Δ*L_b_* were detected during contraction (Fig. 3). These results are most likely explained by differences in both initial fascicle angle and the degree of subsequent rotation along the muscle during contraction. Such differences could affect Δ*L_b,segment_* because it is mathematically equivalent to the cosine of fascicle angle change. MRI data obtained during active lengthening of the human MG show that fascicle lengthening is relatively uniform along the muscle but that fascicle rotation is greater at the proximal than distal muscle end (Shin et al., 2009). Thus, different regions, or segments, of the muscle experience different stretch magnitudes, and spatial variation in segment lengthening is observed. Such behaviour may be muscle length dependent due to differences in fascicle angle and rotation amplitude as the muscle lengthens and possibly explain the length-dependent differences between muscle gear methods. While our results have been confirmed only during active lengthening herein, differences in belly and belly segment gears and their relationship with force or velocity during concentric contractions were previously identified in studies of human bicycling under similar mechanical conditions, although different experimental methods were used to measure muscle length changes (Dick and Wakeling, 2017; Wakeling et al., 2011). Recently, belly gear differences between proximal and distal muscle compartments have been revealed in the rat MG (Tijs et al., 2021) during shortening contractions, and these results along with ours suggest that different compartments of human and animal (Tijs et al., 2021) muscles may play fundamentally different mechanical roles when active force is produced. It would be of interest to determine whether such spatial variation in muscle lengthening occurs during dissipative tasks (e.g. jump landings) in humans and animals of different species that display substantial architectural differences along the muscle.

Differences in belly and segment gears shown herein may be a symptom of initial belly and segment length differences since *L_b,segment_* is much shorter than *L_b_*. Our results show smaller *L_b_* strains than fascicle strains (Fig. 4), so belly strain (εbelly) gears were <1.0 during both active and passive lengthening (Fig. 5C and D), which were much lower than for belly gear (Fig. 5A and B). By contrast, *L_b,segment_* strains were greater than fascicle strains (Fig. 4), resulting in a slightly greater εbelly segment gear. While muscle (segment) gear calculated using strain values was first proposed to quantify salamander muscle mechanics (Azizi and Brainerd, 2007; Brainerd and Azizi, 2005), we believe gear values calculated from strains are nonsensical under the conditions tested herein, i.e., where there is a difference between segment and belly lengths (and their changes) and the same muscle is compared across contraction conditions (Fig. 5C and D). Strain should not be used in muscle gearing studies where whole muscle length change is examined unless *L_b,segment_* and *L_b_* are similar. However, strain can be used when muscle segment length is examined and may be particularly useful when comparing muscles with different shapes. It should also be recognised that some similarity existed in the conclusions drawn from belly gear and εbelly gear measurements.

The finding that MG belly gear downshifts during active lengthening has important functional implications. The high belly gear at short muscle length (plantar flexion) indicates that fascicles lengthened relatively less than the whole muscle, possibly because a larger proportion of muscle length change resulted from fascicle rotation, as evidenced by the larger changes in *L_b_* than *L_b,segment_*. However, the reduction in gear at longer lengths indicates that fascicle lengthening contributes relatively more to muscle lengthening once the muscle is longer, and the fascicles themselves contribute more to mechanical energy dissipation (more negative work was done) as the muscle length increases. In this scenario, muscle fibres may be more susceptible to damage or strain injury (Butterfield and Herzog, 2006; Lieber and Fridén, 1993). Future studies should determine whether muscle gearing behaviour during active lengthening is associated with injury risk during daily or sporting activities, or whether gearing behaviour and thus injury risk is affected by exercise training or detraining.

Overall, the present study showed that substantial differences and inconsistent reporting of muscle gear calculation methods exist between studies. The data presented herein reveal that these methodological differences can produce distinct quantitative and qualitative outcomes that will affect the conclusions drawn from the data, but that these differences may vary between contraction conditions. For example, gear downshifts were observed when *belly gear* but not *belly segment gear* was calculated during active lengthening. However, no gear changes were detected during passive lengthening regardless of the gear computation method used. Gear was higher during active than passive lengthening, with the most substantial differences found at shorter muscle lengths. Fascicle linearity had no meaningful effect on gear calculation under the conditions studied, although further work is required to determine its potential effect in other cases. Simulations may be an interesting avenue to explore such effects. Gear differences during active lengthening found in the present study are thus a symptom of whether the whole muscle length or rather segment changes are computed. The use of whole muscle versus segment length changes might depend upon the specific research question, and they may sometimes completement each other, particularly in cases in which there are substantial differences in architecture between muscle regions or inhomogeneous behaviours of muscle and its passive structures exist during contraction. To estimate the whole muscle belly gear, a measure of whole muscle belly length change in addition to the mean length change of fascicles would be ideal. The latter may be achieved by capturing fascicle length changes at multiple sites along the muscle, allowing for the average fascicle length changes to be used, examination of region-specific differences in fascicle behaviour, and mean whole muscle belly gear to be determined. Input length normalization (i.e. use of strain values) did not affect the conclusions drawn in relation to muscle length-dependent changes (or lack thereof) during lengthening or the differences in gear between contraction conditions, yet εbelly gear values were nonsensical when computed under the current experimental conditions (i.e. where the segment is not representative of the whole muscle). Notwithstanding, the use of strains may be useful when comparing between segments or muscles (particularly those of different shapes) if the segment length change is either the main variable of interest or is representative of the whole muscle. Finally, we conclude that differences in gear nomenclature and methods can affect the gear ratios computed and thus data interpretation, complicating comparisons of results amongst studies. This was particularly demonstrated during maximal active MG lengthening (eccentric) contractions during ankle rotations, and it would be interesting to examine whether muscles with distinct properties, spanning different joints, and performing different contraction types exhibit similar behaviours. We hope the present findings guide future quantification and reporting of ‘muscle gear’ in both animal and human comparative physiology.

## MATERIALS AND METHODS

### Overview

The data used in the present study were collected during a larger study of muscle gearing during muscle lengthening. Participants visited the laboratory on four occasions each separated by ≥72 h. The first two visits were devoted to extensive familiarisation of the test procedures as well as the testing of maximal plantarflexion eccentric contractions while the third and fourth visits required passive and active sub-maximal stretches (eccentric contractions) to be performed on an isokinetic dynamometer. Part of these data, including details of the experimental design and inter-day reliability assessed between the third and fourth visits, have been published previously (Pinto et al., 2021). Here, we used the data collected during both maximal eccentric contractions and passive stretches at a slow (5°/s) velocity, obtained in the second and fourth visits, respectively, to compare different methods of gear calculation. In all sessions, participants performed isometric voluntary contractions at increasing intensities (20–100%) to pre-condition the muscles whilst seated on an isokinetic dynamometer (Biodex System 4, Biodex Medical Systems, Shirley, New York, USA) with the knee fully extended and ankle in the anatomical position (0° plantar flexion). Two maximal voluntary isometric contractions (MVICs) were subsequently performed. After a 30-s rest, participants then had their right ankle rotated into dorsiflexion with the muscles voluntarily relaxed (passive) and then whilst contracting maximally, during which ultrasonography, dynamometry, electromyography, and motion analysis were used to capture the relevant data. Sonography was used to capture images of both the medial gastrocnemius (MG) fascicles and its muscle-tendon junction (MTJ; with Achilles tendon). Along with data captured using motion analysis, this allowed for *L_b_* to be calculated during passive and active joint rotations. From sonograms obtained over the muscle belly, changes in fascicle length, fascicle angle, and muscle thickness were calculated. Belly gear and belly segment gear were derived from these calculations, as described in detail below.

### Participants

Thirteen active men (means±s.d.: age=26.3±5.0 years, body mass=79.2±12.5 kg, height=1.77±0.06 m) free from neuromuscular disease or musculoskeletal injuries and with a minimum 20° dorsiflexion range of motion (ROM) during a slow-velocity ankle stretch (i.e. 5°/s; knee fully extended) volunteered for the present study. Before commencement, participants read and signed an informed consent form and completed a pre-exercise medical screening questionnaire to identify any health condition that would preclude them from performing maximal-effort exercises. Participants refrained from intense exercise within 48 h of testing and avoided the intake of caffeine or alcohol 6 h prior to the testing sessions. All procedures used were approved by the Edith Cowan University Human Research Ethics Committee (ethics project number 19683).

### Dynamometry assessment

Participants were positioned on the chair of an isokinetic dynamometer (Biodex System 4, Biodex Medical Systems, Shirley, New York, USA) with the hip angle at 55° (i.e. semi-reclined), knee fully extended (0°), the ankle in the anatomical position (0°; sole of the foot perpendicular to the shank), and the lateral malleolus aligned to the dynamometer's axis of rotation (Pinto et al., 2019). A rigid clip strap was tightened across the foot to minimise heel displacement from the dynamometer footplate, which was visually confirmed by the investigators of the study prior to warm-up. The participant was seated with knee angle ∼30° flexion before the knee was extended to 0° to take up slack from the dynamometer system (Cannavan et al., 2012). Thereafter, the participant's ankle was rotated at 5°/s from 20° of plantar flexion to full volitional dorsiflexion ROM (point of discomfort at which they could no longer tolerate stretching), with the stretch terminated when the participant pressed a dynamometer control button. Visual feedback of foot rotation was removed by using a cover placed over the thigh during stretches. Participants were asked to completely relax their muscles whilst muscle activity (EMG) feedback was given instantaneously on a screen placed in front of them. For the active (maximal eccentric) contraction trials, the system ROM was set from 20° plantar flexion to 90% of maximum dorsiflexion angle obtained during the passive stretches. Given that variations in maximal ROM existed between participants, analyses were performed through a 40° ROM only (from 20° plantarflexion to 20° dorsiflexion). Ankle torque, joint angle, angular velocity, and EMG data were simultaneously recorded at 2 kHz using Labchart v.8.1.16 Software (Powerlab System, ADInstruments, NSW, Australia). Two to five passive stretches were performed with 1-min inter-trial interval, whereas two to three maximal plantar flexion eccentric contractions were performed with three to five minutes allowed inter trials. The eccentric contraction with the highest peak torque and without significant fluctuation in the torque trace throughout the ROM was analysed. Given that subjects were previously familiarized with the maximal eccentric contractions and the tests required a long time under tension (resulting from slow velocity and large ROM), no more attempts were provided.

### Ultrasonography and architectural calculations

Real-time B-mode ultrasound imaging was used to record MG fascicle behaviour and MG-Achilles MTJ movement during all tests by two independent ultrasound systems. A linear probe (Model UST-5712, 7.5 MHz, B-mode, Aloka, SSD α-10, Japan) with a 50-mm field of view was coated with acoustic coupling gel and oriented along the longitudinal axis of the MG-Achilles MTJ such that the superficial and deep MG aponeuroses could be visualized and the triangulation of the MG-Achilles MTJ was possible (Kay et al., 2016). The ankle was moved through the ROM to check that visualization of the MTJ during trials was possible and, once the location was ascertained, the transducer location was marked on the skin using a surgical marker before fixation. For MG fascicle behaviour, a wide-band linear probe (7.5 MHz, B-mode, Aloka, SSD F-75, Japan) with a 60-mm field of view was placed over the muscle belly (the centre of the image was located ∼50% of the distance from the muscle's origin). When the location was ascertained, the ultrasound transducer was coated with acoustic coupling gel to minimize probe pressure and the transducer was manipulated (tilt, rotation, yaw axes) to ensure optimal fascicle visualization throughout all contractions, i.e. the connective tissues surrounding a bundle of muscle fibres, which appear as individual white echoic lines and are relatively easy to distinguish. Here, we ensured that (i) at least one fascicle (but preferably several) was clearly visually detectable from the start to end of the ankle rotation, and (ii) the superior and inferior aponeuroses were approximately parallel. This was important to ensure accurate estimations of fascicle length change during trials. While it would be ideal to use three-dimensional (3D) ultrasound or other imaging methods, owing to the complex 3D fascicle orientations of the human MG, we were careful to alter the pitch, roll and yaw angle of the probe to best capture the rotation of the fascicles throughout the muscle contraction. It is also pertinent that previous analyses have suggested that probe orientation does not severely affect MG fascicle angle or rotation during active contractions, and it should therefore not substantially impact our gearing estimates (Rana et al., 2013). The ultrasound transducers were fixed on the skin using a stretchable adhesive tape bandage (Elastoband Light, Elastoplast, Australia) holding a custom-made flexible foam cast. During all contractions, real-time ultrasound video images were recorded at sample rates of 25 and 30 Hz (which differed between ultrasound systems) for MG fascicle behaviour and MG-Achilles MTJ, respectively. Ultrasound video, dynamometer data, and EMG acquisitions were synchronized using a 5 V square TTL pulse triggered from the ultrasound into the computer's software (Labchart; ADInstruments, Melbourne, Australia).

The MTJ coordinates were manually digitised frame by frame during both the passive and active trials. The linear distance between the MTJ and the distal edge of the ultrasound was calculated and, in combination with motion analysis, used to estimate whole MG muscle length (*L_b_*; see below). MG fascicle length (*L_f_*) of three observable fascicles were analysed every 5° of ankle ROM during all tests and the mean of the three fascicles was used for analysis. Preliminary fascicle behaviour analyses in one subject revealed negligible differences in results obtained through frame-by-frame analyses (25 Hz sample rate) and that achieved by analysing frames capture at every 5° of the joint ROM (1 Hz sample rate). Thus, the latter method was thus adopted for the full analysis to allow a sufficient number of participants to be tested using manual digitisation methods within a reasonable analysis time frame. Frames of interest were stepped backwards and forwards to ensure the same fascicles were digitised across frames. In pilot testing, we observed that fascicles sometimes projected out of the ultrasound field-of-view, particularly during the passive trials and towards the end of the ROM. In these cases, the non-visible portion of the fascicles was estimated as the length of the line of intersection between the projected fascicle and aponeurosis (based on their visible shape) plus the length of the fascicle path that was directly measured. MG fascicle length was analysed using the segmented line tool using the spline fit function on ImageJ software (National Institute of Health, USA) to follow the curved fascicle path. This fascicular path from the superficial to deep aponeurosis was drawn in the video files, which allowed for retrospective analyses of fascicles, assuming a linear path using a four-point coordinate planimetric model. To do this, the insertions of the fascicles onto the aponeuroses were digitized and fascicle length was defined as the linear distance between the proximal and distal insertions of the drawn fascicles onto the aponeuroses, *L_f,straight_*=straight fascicle length. This is an adaptation of traditionally used methods that assume a linear path of the fascicles (Dick and Wakeling, 2017; Randhawa et al., 2013; Randhawa and Wakeling, 2013; Wakeling et al., 2011). These coordinates also allowed for muscle thickness to be calculated, which was defined as the average (orthogonal) distance between the superficial and deep aponeuroses at the proximal and distal ends of the fascicles (Dick and Wakeling, 2017; Randhawa et al., 2013; Randhawa and Wakeling, 2013; Wakeling et al., 2011). Fascicle angle was subsequently defined as the average angle between the fascicles and the superficial and deep aponeuroses and calculated by trigonometry as 
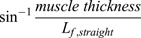
. From these estimates, belly segment length (*L_b,segment_*), also known as ‘projected’ belly length and defined as the length of the fascicle projected onto the muscle's line of action, was calculated as *L_f,straight_*×cos (fascicle angle).

All variables were interpolated using the line of best fit from polynomial fitting to determine the variables of interest at each degree of ROM and to allow for (1) belly gear, Δ*L_b_*/Δ*L_f_*, calculated as the ratio of the change in *L_b_* to the change in *L_f_* (directly measured and thus accounted for fascicle curvature during trials); (2*)* belly segment gear, Δ*L_b,segment_*/Δ*L_f,straight_*, calculated as the ratio of the change in *L_b,segment_* to the change in *L_f,straight_*, both of which were determined from trigonometric calculations assuming fascicle linearity (Randhawa and Wakeling, 2013; Wakeling et al., 2011). To determine the effect of fascicle curvature on belly segment gear, an alternative version of the *belly segment gear* was calculated, named herein as the modified belly segment gear, which was calculated as Δ *L_b,segment_* /Δ*L_f_*. Muscle gear ratios were calculated as a change in variables every 10° of the ROM using the first central difference method and therefore are reported at the following ankle joints ROMs: −10° and −5° of plantar flexion, 0°, and 5° and 10° of dorsiflexion.

While the methods above are, to date, most often used, it should be noted that AGR was first defined as a strain (ε) ratio, i.e., the ratio of longitudinal segment (ε*L_b,segment_*) to fascicle strains (ε*L_f,straight_*) (Azizi and Brainerd, 2007; Brainerd and Azizi, 2005). Thus, all the muscle gear calculations described above were also calculated using strain values (i.e. % change relative to initial length) as inputs to determine whether input normalization affects outcomes. This also accounts for dimension differences between the segment (*L_b,segment_*) and *L_b_* changes to be more appropriately compared. The nomenclatures used were as follows: εBelly gear, εBelly segment gear, and εModified belly segment gear; however, a summary of these nomenclatures and calculations used is included in Table 2.

### Motion analysis

*L_b_* was computed during all tests. One digital camera recording at 25 Hz (Canon Legria, HF M52, Japan) was positioned ∼2 m from the shank and perpendicular to the plane of the leg to record the movement of foot and shank. Reflective markers of 8-mm diameter were placed on the insertion into the Achilles tendon at the calcaneus, the origin of the head of MG at the medial femoral epicondyle, and the distal edge of the ultrasound probe positioned over the MG-Achilles MTJ. Raw coordinate data were sampled at 25 Hz and smoothed using line of best fit from polynomial polynomials. Following previously published methods (Kay et al., 2016), MG *L_b_* was calculated as the distance between the origin of the medial MG head and the marker placed on the distal edge of the probe placed over the MG-Achilles insertion point minus the distance from the actual MTJ position (determined with ultrasound) to the distal edge of the probe.

### Statistical analyses

All analyses were conducted using R (v 3.6.3, R Core Team) in the RStudio environment (v 1.3.1093, RStudio Team). Linear mixed effect models were used to test effect of fascicle length calculation method [two levels: measured directly (assuming curvature; *L_f_*) versus calculated trigonometrically (assumed straight; *L_f,straight_*) on *absolute* fascicle length]. The model was constructed for the active and passive trials with method, ROM (every 5°; nine levels), and their interaction as fixed effects, and participants as a random effect, which allowed intercepts and slopes to vary for the effect of method on muscle gear. Similarly, linear mixed model was used to determine the effect of muscle gear calculation method (belly gear, belly segment gear, and modified belly gear) and whether this effect was dependent on testing condition (passive versus active) and ROM. The model was constructed with method, condition, and ROM (every 5° from −10° plantar flexion to 10° dorsiflexion; five levels), and their interaction as fixed effects, and participants as a random effect, which allowed intercepts and slopes to vary for the effect of method on muscle gear. These analyses were repeated using the strain ratios as the dependent variable. *F* ratios were computed from the analysis of variance. When a significant fixed effect was detected, pairwise comparisons of the estimated marginal means were performed with *P* values adjusted using the Tukey method using the ‘emmeans’ package v 1.5.2.1 (Lenth, 2020). All models were fitted by restricted maximum likelihood using ‘lmerTest’ package v 3.1.2 (Kuznetsova et al., 2017). For visualisation purposes, data are shown as conditional effects (predicted values) and standard errors obtained from the ‘ggpredict’ function of the ‘ggeffect’ package (Lüdecke, 2018). Figures were produced using the ‘ggeffects’ and ‘ggplot2’ (Lüdecke, 2018; Wickham et al., 2016) packages.
